# Disrupted Signalling Landscapes in Glioblastoma: Mechanistic Insights and Therapeutic Frontiers

**DOI:** 10.3390/biology15191689

**Published:** 2026-09-22

**Authors:** Ammar Asif Ali Ansari, Natalia Haddad, Ana Maria Assed, Nada Awaad, Shifa Badruddin, Afroditi Damou, Taslima Haque Shuwara, Marihah Rizwy, Nadine Wehida, Ahmed Elbediwy

**Affiliations:** 1 Department of Biomolecular Sciences, Kingston University London, Kingston-upon-Thames KT1 2EE, UK; k2044983@kingston.ac.uk (A.A.A.A.); k1908666@kingston.ac.uk (N.H.); k2416847@kingston.ac.uk (A.M.A.); nada_02@hotmail.it (N.A.); k2552725@kingston.ac.uk (S.B.); k2551389@kingston.ac.uk (A.D.); k2130887@kingston.ac.uk (T.H.S.); k2252995@kingston.ac.uk (M.R.); 2 Imperial College London, London SW7 2AZ, UK

**Keywords:** glioblastoma, GMB, prognosis, signalling pathways, signalling disruption, clinical therapies, therapeutic interventions, Hippo, ERK/MAPK, PI3K/AKT/mTOR, Wnt/β-catenin, clinical progress

## Abstract

Glioblastoma (GBM) is the most prevalent and aggressive malignant primary brain tumour, originating from glial cells known as astrocytes and accounting for over 50% of all central nervous system malignancies. Its aetiology is linked to various factors, including radiation exposure, genetic alterations, and viral influences, which trigger the uncontrolled growth and rapid infiltration of glial cells into surrounding brain tissues. Despite standard multidisciplinary management involving maximal safe surgical resection, radiotherapy, and temozolomide, the prognosis remains poor. The molecular landscape of GBM is characterised by profound dysregulation across highly conserved signalling networks, including the PI3K/AKT/mTOR, ERK/MAPK, Wnt/β-catenin, and Hippo pathways. These pathways are frequently hijacked by genetic and epigenetic alterations such as EGFR amplification and PTEN loss, which drive essential hallmarks of cancer like uncontrolled proliferation, angiogenesis, and apoptosis evasion. A defining feature of GBM is its extreme intra-tumoral heterogeneity and cellular plasticity, where tumour cells dynamically transition between distinct transcriptional states, including mesenchymal, astrocyte-like, and neural progenitor-like, to adapt to therapeutic pressures. This complexity is further exacerbated by microenvironmental factors. Clinical progress is severely hindered by the blood–brain barrier (BBB), which restricts systemic agents from reaching invasive tumour margins. Targeted monotherapies, such as EGFR and mTOR inhibitors, have largely failed in clinical trials due to compensatory feedback loops and pathway redundancy. Consequently, the therapeutic paradigm is shifting toward rational combination therapies and novel immunotherapeutic platforms. Emerging strategies, such as bivalent CAR T-cell therapy targeting both EGFRvIII and IL-13Rα2, have shown early clinical promise in achieving tumour regression, highlighting a potential path toward precision medicine. This review synthesises current insights into signalling dysregulation and explores the future frontiers of multi-targeted therapeutic interventions in GBM.

## Simple Summary

Glioblastoma is the most common and one of the most aggressive forms of brain cancer, with a poor long-term prognosis because tumours rapidly adapt to standard treatments. This review explores how brain cancer cells hijack internal signalling networks to grow, migrate, and evade normal cellular regulation. We examine why single-gene targeted treatments often fail, as cancer cells switch between pathways, develop resistance, and use various methods to evade and survive both the effects of treatment and the immune system. Additionally, the natural protective barrier between the bloodstream and the brain prevents many drugs from reaching cancer cells deep within brain tissue. Our review highlights how innovative combination strategies can overcome these evolutionary hurdles. Ultimately, shifting towards multi-targeted combination therapies offers a vital path to improving patient survival, offering new hope to individuals and families facing this devastating and often fatal form of cancer.

## 1. Introduction to Glioblastoma

Glial cells are essential components found in the central nervous system (CNS). These cells form the essential non-neuronal infrastructure of the central nervous system, providing structural support, metabolic regulation, synaptic modulation, and immune surveillance. Unlike neurons, glia retains proliferative capacity throughout life, enabling them to respond dynamically to injury, inflammation, and developmental cues.

Glioblastomas (GBM) are a class of tumours that originate in the central nervous system. They arise from glial cells, such as astrocytes, oligodendrocytes, and ependymal cells, which provide structural and metabolic support to the brain and spinal cord [1]. It is among the most aggressive and prevalent types of malignant brain tumour [2]. GBM accounts for over 50% of brain malignancies, with an annual incident rate of approximately 3–4 cases per 100,000 individuals worldwide [3,4,5].

The aetiology of GBM remains largely unknown but is associated with radiation exposure, genetic and lifestyle factors, such as smoking, diet, and alcohol consumption, and viruses, which have been associated to its development. These factors can cause cellular and DNA damage, causing uncontrolled growth of astrocytes, resulting in a tumour that can quickly spread to surrounding brain tissues. They tend to migrate along existing structures in the brain. These include myelinated fibres, the lining of the ventricles, and blood vessels [6].

The clinical presentation of GBM varies according to tumour location, size, and extent of infiltration. Patients may experience headaches, seizures, cognitive or behavioural changes, or focal neurological deficits involving motor, sensory, language, or visual function. Symptoms of raised intracranial pressure, including headache, nausea, and vomiting, may also occur in patients with substantial tumour growth or oedema [7].

Surgery is usually recommended for the most accessible primary tumours, provided it can be performed without causing neurological damage to the patient. Surgical resection is often accompanied by radiotherapy to ensure complete removal of the tumour. It requires precise shielding of sensitive structures, such as the eyes and oral cavity, to minimise toxicity. In cases of metastatic spread, whole-brain radiotherapy is performed. Chemotherapy is limited by the blood–brain barrier, which prevents many drugs from reaching the tumour. However, certain drugs (e.g., methotrexate or cytarabine) can be administered intrathecally via lumbar puncture into the cerebrospinal fluid [8,9].

Despite various therapeutic interventions, the prognosis of GBM remains poor. Median overall survival is typically 12–15 months, with fewer than 10% of patients surviving beyond five years [10].

This limited response stems from the tumour’s rapid growth and highly infiltrative nature, characterised by its invasive cancerous extensions into adjacent brain tissue, which makes complete surgical removal nearly impossible [11]. Additionally, the blood–brain barrier restricts drug delivery, and intrinsic resistance mechanisms further reduce treatment efficacy [12].

The pathogenesis of GBM involves the dysregulation of various cellular signalling pathways, which regulate fundamental biological processes such as cell proliferation, survival, apoptosis evasion, invasion, and angiogenesis. These pathways, including classical cancer-related pathways, include PI3K/AKT/mTOR, MEK/ERK, p53, Notch, Wnt, Hedgehog, and Hippo, all of which form interconnected networks that respond to extracellular stimuli and maintain cellular homeostasis under normal conditions. In malignancies, genetic alterations hijack these pathways, causing tumour progression [13]. Understanding these signalling pathways is crucial, as they not only give insight into the molecular mechanisms of cancer but also help identify therapeutic targets and enable precision medicine.

With such a complex clinical condition, this review aims to explore GBM at a molecular level, expanding on signalling pathways and their impact on the tumour microenvironment, the treatment challenges of the blood–brain barrier (BBB), and comprehensive molecular disruptions, and understand current therapeutic novelties and their contribution in improving prognosis, outcome, and patient care. With over 100 studies being used in the development of this review, it will dive into current therapeutic barriers and treatment insights in seven sections, breaking down the mechanisms involved and how new trials and novel treatment approaches may impact care.

## 2. Cellular Signalling

In recent years, research has highlighted that dysregulated signalling pathways contribute to the pathogenesis of aggressive cancers such as GBM. These aberrations often arise from molecular alterations, including, for instance, the overexpression or loss of critical regulatory genes, which disrupt normal cellular fate and structure. Such genetic changes drive malignant behaviour in GBM, underscoring the need to understand the underlying key signalling pathway that contributes to the development of this cancer in GBM patients.

Several core signalling cascades have emerged as key drivers of GBM’s aggressive phenotype. These include the extracellular signal-regulated kinase 1/2 mitogen-activated protein kinase (ERK1/2-MAPK) pathway, the phosphatidylinositol 3-kinase (PI3K)/Akt pathway, the p53 tumour suppressor network, the Wnt/β-catenin signalling axis, and the Hippo pathway [14,15,16]. Aberrant activity in each of these pathways has been strongly associated with the development of therapeutic resistance and with unfavourable patient outcomes in GBM [17]. For instance, it has been reported that molecular changes associated with PI3k/Akt pathway (increased expression of epidermal growth factor receptor (EGFR) or loss of PTEN) are associated with 88% of GBM cases [18]. Likewise, genetic alterations in the p53 complex and aberrant activation in the WNT signalling pathway are associated with 84% prevalence and 12–18-month median survival post-diagnosis, respectively [19,20].

As these pathways regulate essential cellular functions, including proliferation, repair, and apoptosis, understanding how these conserved networks drive GBM is vital for the development of effective therapeutic strategies.

### 2.1. MAPK and ERK Signalling Pathway

The ERK/MAPK pathway plays a fundamental role in translating extracellular signals responsible for regulating crucial cellular processes associated with cell proliferation and differentiation. This pathway governs critical biological processes, including differentiation and survival in response to external cues like stress, heat shock, and inflammatory cytokines. The MAPK pathway is an intracellular signalling network activated by cell surface receptors and various stress-related stimuli. The mitogen-activated protein (MAP) kinase (MAPK) cascade consists of a core three-tier kinase comprising MAP3K, MAPKK, and MAPK. In response to specific stimuli, two additional kinases also get recruited, i.e., MAP4Ks upstream of MAP3Ks and MAPK-activated protein kinases (MAPKAPKs) downstream of MAPKs, thereby forming a five-tier signalling cascade [21]. Sequential phosphorylation and activation of these kinases enable the transmission of extracellular signals to downstream cellular targets. Activated MAPKs can phosphorylate cytoplasmic substrates and translocate to the nucleus, where they regulate transcription factors and alter gene expression to generate specific cellular responses [22,23]. This signalling cascade is illustrated in Figure 1.

The ERK1/2 signalling pathway can be activated following stimulation of several classes of cell surface receptors, including receptor tyrosine kinases (RTKs) and G-protein-coupled receptors (GPCRs). Receptor activation promotes activation of the small GTPase RAS at the plasma membrane, which subsequently recruits and activates RAF proteins. RAF proteins function as MAP3Ks and phosphorylate MEK1/2, which act as MAPKKs. Activated MEK1/2 subsequently phosphorylate ERK1/2 on conserved threonine and tyrosine residues within their activation motifs [24]. Once activated, ERK1/2 phosphorylates numerous cytoplasmic substrates and can translocate to the nucleus, where it regulates proteins involved in cellular processes including proliferation, survival, differentiation, and morphological responses [25].

**Figure 1:**
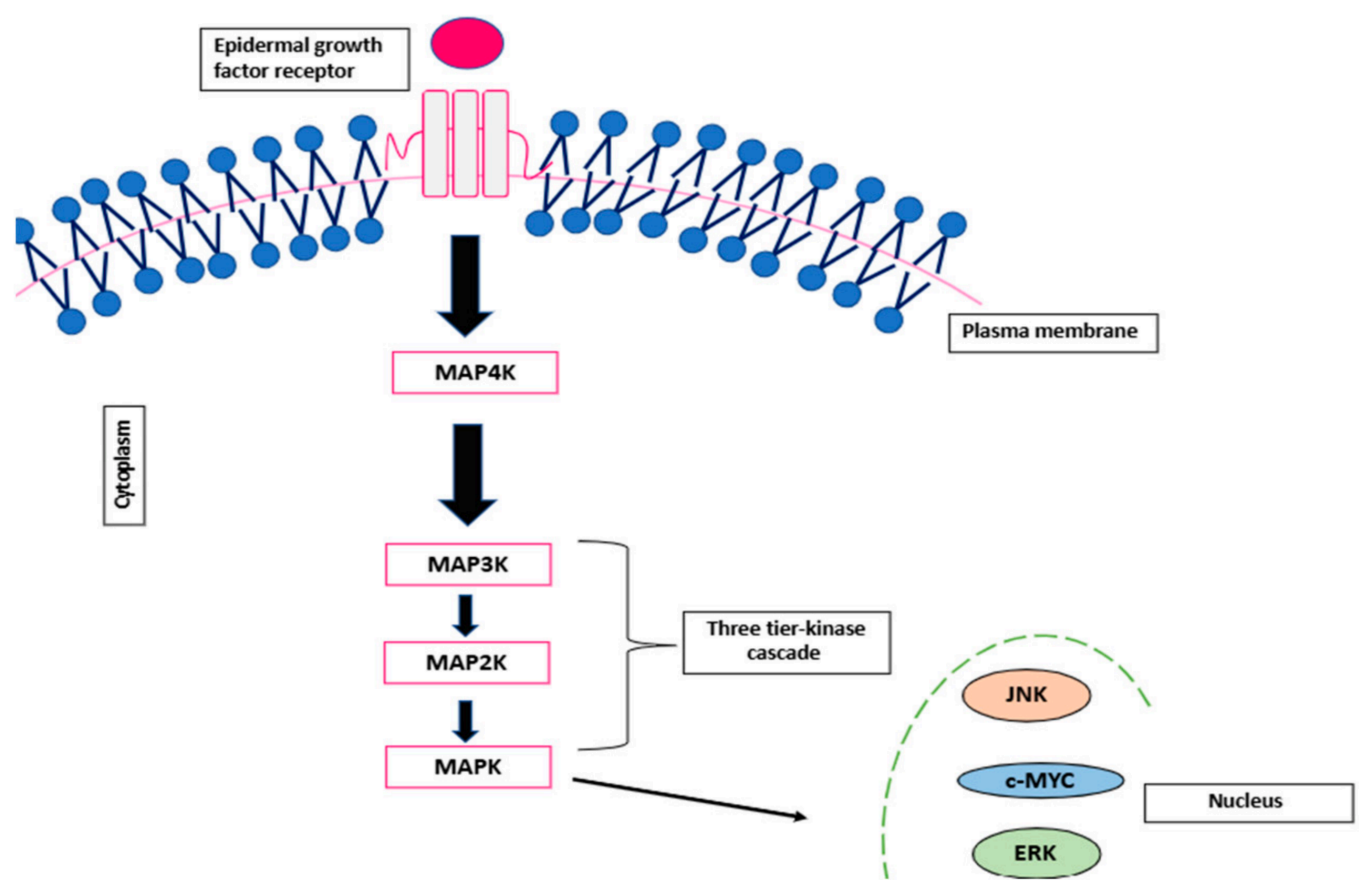
Schematic representation of the ERK/MAPK signalling cascade. Activation of cell surface receptors, illustrated here by the epidermal growth factor receptor (EGFR), initiates a sequential MAPK phosphorylation cascade in the cytoplasm. Upstream MAP4K/MAP3K activity promotes activation of MAP2K, followed by MAPK, forming the core three-tier kinase module. Activated MAPKs, including ERK and JNK family members, subsequently regulate nuclear substrates and transcription factors such as c-MYC, thereby modifying gene expression associated with cell proliferation, survival, differentiation, and morphological responses to extracellular stimuli. Within the classical ERK1/2 pathway, receptor activation is typically transmitted through RAS–RAF (MAP3K)–MEK1/2 (MAP2K)–ERK1/2 (MAPK), after which phosphorylated ERK1/2 translocate to the nucleus to regulate downstream transcriptional programmes. Taken from [26].

### 2.2. PI3K/AKT Pathway

The PI3K/AKT/mTOR signalling cascade is a highly conserved intracellular pathway composed of lipid kinases that govern fundamental cellular functions, including proliferation, growth, and survival. Beyond these roles, it acts as a master regulator by integrating signals from a variety of upstream stimuli and coordinating with other intracellular signalling networks to maintain cellular homeostasis and respond to environmental cues [18].

PI3K enzymes are categorised into three main classes, Class I, II, and III, based on their structural characteristics and substrate preferences. Among them, Class I PI3Ks are most prominently implicated in oncogenesis. This class comprises a catalytic subunit (p110) and a regulatory subunit (p85), which become activated in response to upstream signals from receptor tyrosine kinases or G-protein-coupled receptors (GPCRs), initiating a cascade of downstream signalling events [27].

Once activated, the p110 subunit catalyses the phosphorylation of phosphatidylinositol-4,5-bisphosphate (PIP2) to generate phosphatidylinositol-3,4,5-trisphosphate (PIP3), a crucial secondary messenger. PIP3 then recruits key downstream kinases such as AKT and PDK1 to the plasma membrane by binding to their pleckstrin homology (PH) domains, enabling their activation and subsequent promotion of cell survival and growth signals [28]. This signalling architecture is illustrated in Figure 2.

### 2.3. The Wnt/β-Catenin Signalling Pathway

The Wnt/β-catenin pathway is a highly conserved and complex signalling system that plays a pivotal role in regulating essential cellular functions such as proliferation, differentiation, migration, angiogenesis, and tissue maintenance. This pathway is initiated through interactions between a family of 19 cysteine-rich Wnt glycoproteins and specific membrane-bound receptors and ligands, triggering a cascade of events that ultimately control the localisation and phosphorylation status of β-catenin, a key transcriptional co-activator involved in the regulation of genes linked to pluripotency, cell cycle progression, and metabolic activity [29].

Wnt signalling operates through two main branches: the β-catenin-dependent (canonical) pathway and the β-catenin-independent (non-canonical) pathway. In the canonical route, binding of Wnt ligands such as WNT-1 to Frizzled (Fz) receptors and co-receptors LRP5/6 on the cell surface triggers recruitment of Dishevelled (Dsh) to the membrane. This leads to disassembly of the β-catenin destruction complex, which includes APC, Axin, GSK3β, CK1α, and PP2A. Once signalling is disrupted, β-catenin escapes phosphorylation, accumulates in the cytoplasm, and translocates to the nucleus. There, it partners with transcription factors of the TCF/LEF family to drive the expression of target genes, including c-MYC, c-JUN, and Cyclin D1 [30]. An overview of this process is illustrated in Figure 3.

### 2.4. The Hippo Signalling Pathway

Originally discovered in Drosophila melanogaster, the Hippo signalling pathway is an evolutionarily conserved regulatory network critical for controlling tissue growth, organ size, and cellular regeneration processes [31]. Its core components include the Ste20-like kinases MST1/2, large tumour suppressor kinases LATS1/2, scaffold proteins SAV1 and MOB1, and the transcriptional co-activators Yes-associated protein (YAP) and TAZ (also known as WWTR1). The canonical signalling cascade centres on the phosphorylation status of YAP and TAZ. When phosphorylated, these co-activators are retained in the cytoplasm or targeted for proteasomal degradation. When dephosphorylated, they translocate into the nucleus, where they bind TEAD transcription factors, initiating gene programs that drive cell survival and proliferation [32].

Activation of the Hippo pathway can be triggered by intrinsic cues, such as cell polarity, adhesion, and cell–cell contact, or external mechanical and biochemical signals. Upon stimulation, MST1/2 kinases form a complex with SAV1, facilitating the activation of LATS1/2 through MOB1. Activated LATS1/2 then phosphorylate YAP and TAZ, leading to their cytoplasmic sequestration via interaction with 14-3-3 proteins or their subsequent degradation. This regulatory cascade is depicted in Figure 3A.

However, when Hippo signalling is inactive (“OFF” state), YAP and TAZ escape phosphorylation and accumulate in the nucleus. There, they associate with TEAD family transcription factors to activate genes that inhibit apoptosis and promote cell cycle progression and tissue growth [32]. This nuclear signalling axis is illustrated in Figure 4.

## 3. Mechanism of Dysregulation in Glioblastoma

Dysregulation in GBM and cancer in general is a complex process involving mutations, epigenetics, and cellular metabolic dysfunction, which lead to a collapse of homeostasis in the cell, resulting in neoplasia. These processes work simultaneously with other cellular systems by evading the immune system through disruption of signalling checkpoints and by hijacking surrounding healthy cells, which facilitates tissue invasion. These mechanisms are part of the hallmarks of cancer; a heuristic approach aims at simplifying complex oncogenic mechanisms to better understand and analyse similar genetic and phenotypic features observed across different cancers [33].

Recent research suggests that aggressive progression, signalling disruption, and therapeutic resistance tend to be the most common mechanisms. These include EGFR amplification, unmethylated O6-methylguanine-DNA methyltransferase and histone epigenetic modifications (e.g., methylation and acetylation), mitophagy dysregulation (e.g., SNX7 gene), apoptosis (RNA splicing), and IDH alterations [34,35]. These, collectively, influence the rate of cell proliferation, growth, and self-renewal capacity, resulting in a more resilient tumour capable of surviving extreme conditions such as hypoxic environments and withstanding aggressive treatments, i.e., chemotherapy [13,36].

In addition, multiple signalling pathways have also been shown to be closely associated with GBM’s development, diagnosis, and prognosis, thus assisting with the development of therapeutic targets and a personalised approach to treatment [37]. Activation of the PI3K/AKT/mTOR pathway, for instance, which was observed in 70% of GBM patients, promotes tumour cell survival, proliferation, and metabolic reprogramming, thereby increasing tumour growth and resistance. Similarly, the Wnt/β-catenin pathway inhibits cell differentiation, which maintains the tumour stem cell population. This pathway is further regulated by epigenetic alterations, such as hypermetilation of inhibitors. It also plays an important role in tumour grading and prognosis by analysing the overexpressed Wnt ligands (Wnt2, Wnt3a, and Wnt5a) and β-catenins [13].

Hippo/Notch signalling has been recently linked to tumorigenesis and metastasis in major cancers, including GBM. From regulating size, apoptosis, and proliferation to influencing the functioning of other pathways (negatively regulating AKT activity), it demonstrates perfectly how complex normal system hijacking occurs in glioblastoma. Studies reveal that YAP/TAZ are closely related to its poor prognosis, showing TAZ-induced necrosis and chemoresistance and poor immunological response to the tumour [13,37,38].

Neoplastic cells often cause dysregulation in the PD-1/PD-L1 axis, which regulates programmed cell death and represents major checkpoint molecules [39,40,41]. In GBMs, these dysregulations include the promotion of a pro-inflammatory tumour microenvironment in which cytokines (e.g., IL-6) infer PD-L1 expression in bone marrow, inhibiting T-cell activation and ultimately generating T-cell immunity, which evades natural immunological reaction towards the tumour [42]. The capacity of intracranial tumours to sequester naive T-cells from bone marrow, a tumour-adaptive T-cell dysfunction using CD4+ reprogramming, has also been studied [43].

## 4. Tumour Heterogeneity and Plasticity

Tumour heterogeneity and plasticity are key features underlying the morphological and biological complexity of GBM. Tumour heterogeneity can be broadly classified into inter-tumoral heterogeneity, which refers to differences between tumours from different patients, and intra-tumoral heterogeneity, which describes variability within a single tumour. In GBM, epigenetic dysregulation plays a major role in enhancing spatial intra-tumoral heterogeneity, resulting in distinct transcriptional and genetic profiles across different tumour regions. Multiple studies have demonstrated that individual GBM tumours contain genetically distinct subclones with diverse morphologies and behaviours. Variations in DNA methylation, histone modifications, and chromatin accessibility contribute to heterogeneous gene expression programs that support a range of cellular phenotypes, including stem-like, proliferative, and invasive states [44].

Furthermore, GBM tumours often comprise cells corresponding to multiple molecular subtypes, with one subtype typically predominating, thereby making each tumour biologically unique and particularly challenging to treat. Spatial genetic and epigenetic diversity has been further characterised using three-dimensional neuron navigation-guided sampling, revealing that genomic and epigenetic alterations can differ significantly between regions of the same tumour [45]. In parallel, high-throughput proteomics studies assessing protein–protein expression patterns across large GBM cohorts have identified substantial inter-patient diversity, leading to the classification of tumours into numerous subgroups, while some patients do not fit neatly into any category due to unique protein network alterations.

Consistent with these findings, recent investigations have shown that GBM biomarker expression is not homogeneous, highlighting variability both between patients and within individual tumours. Notably, Sottoriva et al. demonstrated that spatially distinct fragments from a single GBM tumour could be assigned to different molecular subgroups based on genomic analysis [46]. In their study, one of the subclones exhibited amplification of EGFR, CDK6, and MET, whereas another subclone acquired a copy of chromosome 3 containing PIK3CA, resulting in PIK3CA amplification, underscoring the profound intra-tumoral heterogeneity characteristic of GBM.

The presence of multiple, coexisting tumour cell subpopulations underlies the plasticity of glioblastoma, enabling tumour cells to adapt dynamically to therapeutic pressure and thereby develop resistance to treatments such as receptor tyrosine kinase (RTK) inhibitors and radiotherapy. Rather than representing fixed lineages, GBM cells occupy a limited number of transcriptional states, including oligodendrocyte precursor-like, neural progenitor-like, astrocyte-like, and mesenchymal, all of which retain proliferative capacity. Tumour plasticity arises from the ability of cells to transition between these states, allowing the tumour to maintain growth despite selective pressures, while one state typically predominates within a given tumour due to underlying genetic alterations [47].

Genetic drivers strongly bias state dominance, with EGFR amplification favouring the AC-like state, CDK4 amplification promoting NPC-like programs, and PDGFRA alterations enriching OPC-like populations. In contrast, the mesenchymal state is closely associated with inflammatory cues from the tumour microenvironment, particularly interactions with macrophages and microglia. Ligand-mediated signalling and microenvironmental factors further regulate transitions into and out of the mesenchymal state, highlighting the dynamic and reversible nature of these cellular programs [48].

Furthermore, cancer stem cells (CSCs), which are self-renewing tumorigenic cells, play a crucial role in GBM heterogeneity and tumour recurrence. GSCs are multipotent and can differentiate along multiple lineages into progenies with varying characteristics, creating distinct niches that heavily contribute to the heterogeneity observed in GBM [49]. Specifically, GSCs drive GBM progression through multiple mechanisms, including immune suppression, restricting immune cell access to nutrients such as glucose and oxygen, promoting angiogenesis, and enhancing invasion and metastatic potential. Additionally, GSCs exhibit greater infiltration and migratory capacities through engagement in the epithelial–mesenchymal transition (EMT) pathway. By leveraging these characteristics, GSCs can shape a supportive tumour microenvironment (TME), further promoting cancer progression and worsening disease outcomes [50].

Research has demonstrated that protein expression in GSCs is highly diverse, reflecting the heterogeneity of the disease. Tumour markers such as CD133 are commonly overexpressed in GSCs; however, studies have shown that even CD133-negative cells can exhibit comparable levels of tumorigenesis in GBM, regardless of their plasticity index [51].

Soeda et al. demonstrated that single cells derived from one patient can produce phenotypic and self-renewal differences both in vitro and in vivo [52]. Similarly, Berezovsky et al. suggested that the capacity of tumour cells to mutually interconvert is influenced by genetic, epigenetic, and microenvironmental regulation, allowing GBM cells to alter their phenotypic and functional roles, thereby contributing to tumour growth [53]. These two studies provide strong evidence regarding both inter-tumour and intra-tumour heterogeneity, proving the importance of the adaptive transitions and implications of GCS in the development of effective treatment.

## 5. Therapeutic Strategies in GBM

The current standard treatment for GBM typically involves maximal safe surgical resection, often combined with radiotherapy and temozolomide-based chemotherapy. However, durable disease control remains challenging. Radical surgical removal is not feasible due to the highly infiltrative nature of GBM within the brain parenchyma, and residual infiltrative tumour cells can contribute substantially to the high rate of reoccurrence [54,55].

A major challenge in the treatment of GBM is the blood–brain barrier (BBB), which is formed by specialized endothelial cells lining the cerebral microvasculature and supported by tight junction and active efflux mechanisms mediated by transporters such as P-glycoprotein, which restricts the penetration of many systemic therapies into the brain. Although the BBB may be disrupted within parts of the tumour core, vascular permeability is spatially heterogeneous, which causes uneven drug distribution [56]. Consequently, some chemotherapeutic and targeted agents may fail to achieve therapeutically effective concentrations throughout the tumour, thereby limiting treatment efficacy [57].

In parallel with these clinical challenges, large-scale genomic and molecular profiling studies have identified recurrent genetic alterations and dysregulated signalling pathways that drive GBM growth and survival [58]. These discoveries have provided a strong biological rationale for the development of therapies targeting molecular drivers, such as receptor tyrosine kinases (RTKs), epidermal growth factor receptor (EGFR), and downstream signalling cascades, like the PI3K/AKT/mTOR pathway [59]. While these approaches have demonstrated promising results in preclinical models, their translation into meaningful clinical benefit has been limited [18].

The limited efficacy of targeted therapies in GBM reflects the complex interplay between tumour heterogeneity, pathway redundancy, adaptive resistance mechanisms, and restricted drug delivery imposed by the BBB. As a result, inhibition of a single molecular target is often insufficient, as tumours can activate compensatory mechanisms that bypass pathway blockade. Thus, comprehensive and combined therapeutic strategies are required to maximise the potential of pathway-targeted approaches.

### 5.1. EGFR Inhibitors

Among the dysregulated signalling pathways identified in GBM, abnormal activation of receptor tyrosine kinase family members, particularly EGFR, has been one of the most extensively investigated therapeutic targets.

EGFR is among the most frequently altered genes in GBM, with amplification, overexpression, and activating mutations such as EGFRvIII observed in approximately 33.3% of GBM patients [60]. These alterations established EGFR as an attractive therapeutic target and prompted extensive clinical investigation of small-molecule tyrosine kinase inhibitors (TKIs), including erlotinib and gefitinib. Although these agents effectively suppress EGFR signalling in other malignancies, their clinical performance in GBM has been largely disappointing [61].

Importantly, the lack of biomarker-driven patient stratification in many early clinical trials likely contributed to therapeutic failure, as EGFR amplification alone does not reliably predict pathway dependency or treatment response [62].

The overall lack of durable clinical benefits from EGFR-targeted TKIs reflects the biological complexity of GBM. Intratumoral heterogeneity of EGFR expression, the presence of EGFR variants with reduced sensitivity to TKIs, and compensatory activation of alternative receptor tyrosine kinases and downstream signalling pathways all contribute to therapeutic resistance. Furthermore, loss of PTEN function and constitutive activation of the PI3K/AKT pathway can uncouple tumour growth from EGFR dependency, further limiting the effectiveness of EGFR inhibition [13]. These factors underscore the challenges of single-agent EGFR-targeted therapy in GBM and highlight the need for more precise, combination-based therapeutic strategies. Additionally, EGFRvIII expression is spatially heterogeneous within GBM tumours, resulting in incomplete target coverage and further limiting the efficacy of EGFR-targeted TKIs. These limitations have driven the development of alternative strategies, including monoclonal antibodies and antibody–drug conjugates designed to target glioblastoma-specific EGFR conformations [63].

### 5.2. mTOR Inhibitors

Given the frequent dysregulation of the PI3K/AKT/mTOR signalling pathway in GBM via loss of PTEN activity or upstream RTK activation, mTOR complex (mTORC) inhibitors have been extensively investigated as potential therapeutic agents. First-generation mTORC1 inhibitors, such as everolimus, ridaforolimus, and temsirolimus, were developed to suppress mTORC1-dependent signalling, while subsequent generations of ATP-competitive mTOR kinase inhibitors have been designed to achieve broader inhibition of mTOR signalling [64].

A major limitation of these first-generation mTORC1 inhibitors is the activation of compensatory feedback loops upstream PI3K and AKT signalling, which can restore proliferative and survival pathways and contribute to therapeutic resistance [65].

To overcome this, second- and third-generation mTOR inhibitors have been developed, including ATP-competitive mTOR kinase inhibitors such as INK128, Torin 1, and AZD8055. These agents are designed to comprehensively suppress mTOR signalling and target resistance-associated mutations. Preclinical studies have demonstrated improved pathway inhibition; however, their clinical translation remains limited by inadequate BBB penetration, restricting effective drug delivery [66].

### 5.3. Chimeric Antigen Receptor (CAR) T-Cell Therapy

CAR-T-cell therapy involves engineering autologous T-cells to express chimeric antigen receptors targeting GBM-associated antigens such as EGFRvIII, IL-13Rα2, HER2, and EphA2. Early clinical studies have demonstrated CAR-T-cell bioactivity and tumour regression in selected patients. However, the efficacy of single-antigen CAR-T-cell approaches is limited by intratumoral antigen heterogeneity and antigen loss, which can promote immune escape and disease recurrence [67].

Consequently, CAR-T-cell therapy in combination with targeted therapies or immune checkpoint inhibition is being investigated to enhance T-cell activity and overcome resistance mechanisms in GBM. Systemically administered CAR-T cells may encounter limitations in trafficking to intracranial tumours, while the immunosuppressive GBM microenvironment can impair T-cell persistence and promote functional exhaustion. Combination therapies therefore aim to improve CAR-T-cell efficacy by modifying the tumour microenvironment, disrupting immunosuppressive signalling, and enhancing T-cell structural and functional integrity [68].

One approach under investigation combines B7-H3-targeted CAR-T-cell therapy with temozolomide. B7-H3 is expressed in approximately 70% of tumours while exhibiting limited or low-level expression in healthy tissue, making it a potential tumour-associated target for CAR-T-cell therapy. The study used patient’s autologous T-cells, which were then activated and transduced with retrovirus containing B7-H3 CAR-T. The study plans to administer three injections at two dose levels with an interval of 1–2 weeks between Temozolomide cycles [69].

A strategy that has been investigated involves combining CAR-T-cell therapy with immune checkpoint blockade to counteract CAR-T-cell exhaustion. Approaches include the co-delivery of checkpoint inhibitors and the genetic engineering of CAR-T cells to inhibit interactions between checkpoint receptors and their ligands. CAR-T cells can also be engineered to secrete checkpoint blocking agents locally within the tumour microenvironment, which may increase their local concentration while potentially reducing systemic exposure and improving anti-tumour activity [70].

Armoured or antibody-receptor-modified CAR-T-cell approaches have also been developed to enhance activity within the immunosuppressive tumour microenvironment. For example, EGFRvIII-targeted CAR-T cells engineered to locally secrete anti-PD-1 antibodies have demonstrated enhanced tumour control in orthotopic GBM models by counteracting local immunosuppressive signalling [71].

### 5.4. Combination Therapy

Limited efficacy of monotherapies in GBM is primarily attributed to tumoural heterogeneity. GBM tumours can contain multiple molecular and transcriptional states, such as proneural, classical, or mesenchymal programmes. These distinct states are characterised by unique profiles and signalling dependencies. For instance, the classical subtype is closely linked to EGFR-driven signalling, while the mesenchymal phenotype is associated with enhanced cellular invasion, inflammatory response, and therapeutic resistance. Similarly, GBM cells exhibit high phenotypic plasticity, allowing them to transition between these states. This dynamic shifting enables tumour populations to adapt to and bypass pathway-specific treatments [72]. This cellular adaptability is driven by glioblastoma stem-like cells (GSCs), which maintain the tumour’s capacity to evade treatment [73].

The tumour microenvironment can also express multiple molecules such as tenscin-C, a glycoprotein that enhances the invasive nature of glioblastoma cells by utilising non-adhesion and focal adhesion kinase pathways. Fibulin-3 is another type of these soluble glycoprotein cells, which promote growth, invasion, chemoresistance, and survival of the endothelial cells, astrocytes, and GSCs via both Notch- and NF-kB-dependent mechanisms. Additionally, exosomes contain fusion proteins can promote mesenchymal transformation and invasion of GBM [74]. Oral alkylating agents like temozolomide (TMZ) have been used as the primary chemotherapy treatment alongside radiotherapy in the standard Stupp protocol for GBM. It exerts its cytotoxicity by methylating DNA, leading to mismatch repair and, ultimately, detrimental DNA damage [75]. However, its effectiveness has been compromised by both innate and acquired TMZ resistance [76]. Similarly, targeted therapies against the RTK/RAF/MEK/ERK pathway have proven to have limited efficacy despite being considered a key driver in GBM proliferation and survival [77]. This was predominately due to the reactivation of this pathway or alternative signaling pathways in compensation, thus causing resistance to apoptosis and modifying the tumour microenvironment, along with epigenetic reprogramming further contributing to this resistance [78].

Consequently, overcoming these challenges has caused rational combination therapies to emerge as an essential paradigm for battling GBM. In combination therapies, multiple drugs are used simultaneously to attack the tumour, whether by cytotoxicity, blocking pathway functions, or reversing immunosuppression [79]. Throughout this section, we will discuss key combinations that indicate the importance of combination therapy in modern neuro-oncology.

### 5.5. Nanomedicine Therapy

GBM vasculature is frequently characterised by abnormal and heterogeneous permeability. However, infiltrative glioma cells can extend into surrounding brain parenchyma where the BBB remains relatively intact, thereby limiting the delivery of systemically administered therapeutics. Nanocarriers have therefore been investigated as a means of improving drug transport across the BBB through several mechanisms. One widely studied approach is receptor-mediated transcytosis (RMT), in which nanoparticles are functionalised with ligands that bind to receptors expressed on brain endothelial cells and exploit the transcellular transport pathways. Common targets include the transferrin receptor (TfR), low-density lipoprotein receptor-related protein 1 (LRP1), which can be targeted using ligands such as Angiopep-2, and the insulin receptor [80].

Liposomes and lipid nanoparticles (LNPs) are a different class of nanoparticle known for biocompatibility and their ability to encapsulate a wide range of therapeutic cargo [81]. They can be utilised to transport chemotherapeutic agents or nucleic-acid-based therapies and protect them during systemic circulation. LNPs are particularly useful, as their surfaces can be modified with polyethylene glycol (PEG) to prolong circulation time and with targeting ligands to enhance BBB transport and uptake by glioblastoma cells [82].

Polymeric nanoparticles represent another important class of nanocarriers being investigated for drug delivery in GBM. Polymeric nanoparticles are formed from biodegradable or biocompatible polymers that can encapsulate therapeutic agents and gradually release them after administration [83]. Commonly used materials include poly(lactic-co-glycolic acid) (PLGA), polyethylene glycol (PEG), and chitosan. PLGA is particularly useful because it can degrade into relatively non-toxic by-products and designed to release its therapeutic cargo over a prolonged period, thereby helping to maintain drug concentrations within the tumour while potentially reducing systemic exposure [84].

Inorganic and metallic nanomaterials, such as superparamagnetic iron oxide nanoparticles (SPIONs), gold nanoparticles (AuNPs), and silica-based nanoparticles, possess distinctive physical, electronic, and magnetic properties that make them particularly suited for theranostic applications [85]. SPIONs typically consist of an iron oxide core (magnetite, ${\mathrm{Fe}}_{3}O_{4}$, or maghemite, $\gamma {\text{-}\mathrm{Fe}}_{2}O_{3}$) coated with biocompatible polymers such as dextran or PEG [84,85]. They utilize their superparamagnetic properties to act as contrast agents in $T_{2}/T_{2}^{*}$-weighted magnetic resonance imaging, clearly outlining infiltrative tumours [86,87].

Beyond diagnostic imaging, SPIONs can be used for magnetic fluid hyperthermia. When exposed to an alternating magnetic field, they convert magnetic energy into heat via Néel and Brownian relaxation, reaching therapeutic temperatures between approximately 42 °C and 45 °C that induce localized apoptosis and sensitize radioresistant GBM cells whilst minimizing damage to adjacent healthy brain tissue [88,89]. High-atomic-number-based nanoparticles like gold nanoparticles (Z=79) provide dual functionality as contrast agents for computed tomography and radiosensitisers for clinical radiotherapy [90,91]. Because their mass energy absorption coefficient is substantially higher than that of soft tissue, gold nanoparticles can absorb incident radiation photons and release intense cascades of short-range secondary electrons, specifically low-energy photoelectrons and Auger electrons that generate lethal clusters of reactive oxygen species, causing double-strand DNA breaks specifically within adjacent glioma cells [92,93].

## 6. Clinical Translation and Therapeutic Challenges

Despite extensive preclinical investigation of molecularly targeted therapies in GBM, translation into substantial and durable clinical benefit has remained limited. Clinical studies have highlighted several recurrent barriers, including intratumoural heterogeneity, inadequate patient stratification, compensatory pathway activation, restricted drug delivery across the blood–brain barrier, and treatment-associated toxicity.

Clinical trials evaluating erlotinib have failed to demonstrate a meaningful improvement in overall survival even when combined with standard therapies such as temozolomide, radiotherapy, or other synergistic agents [94]. These limited outcomes are likely attributable, at least in part, to the inability of erlotinib to effectively target glioblastoma-specific EGFR alterations, which predominantly affect the extracellular domain rather than the intracellular kinase domain for which these inhibitors were designed. Gefitinib has shown evidence of tumour penetration and target engagement results in select studies, with modest improvements in median survival and proof of substantial drug accumulation within resected glioblastoma tissue. Molecular analyses of patient samples confirmed effective dephosphorylation of EGFR, indicating successful target engagement. However, gefitinib monotherapy was insufficient to fully suppress downstream oncogenic signalling or prevent tumour progression [95].

Everolimus has been evaluated in Phase II clinical trials in GBM patients; however, no significant improvement in progression-free survival (PFS) was observed compared with control groups. Notably, treatment was associated with increased toxicity, highlighting a limited therapeutic window without clear clinical benefit [96,97]. Similarly, ridaforolimus was assessed in a peri-surgical Phase I study, but the trial was discontinued due to slow patient accrual and logistical challenges related to drug administration following surgery, preventing definitive conclusions regarding its efficacy [98]. Temsirolimus has also been investigated in Phase II trials, where it demonstrated modest clinical results. Median overall survival was reported at 14.8 months, compared with 16.0 months in control groups, while median PFS reached 5.4 months versus 6.0 months in controls. These findings suggest that temsirolimus does not confer a survival advantage when used as a single-agent therapy in GBM [99].

More recently, CAR-T-cell strategies incorporating dual-antigen specificity, alternative delivery routes, and modulation of the tumour microenvironment have entered clinical investigation. A Phase I study by Bagley et al. (2025) evaluated intracerebroventricular bivalent CAR-T cells targeting EGFR epitope 806 and IL-13Rα2 in patients with recurrent EGFR-amplified glioblastoma [100]. Among 13 patients with measurable disease, 8 (62%) demonstrated radiographic tumour regression, including one patient with a confirmed partial response and another with durable stable disease lasting more than 16 months. Grade 3 neurotoxicity occurred in 56% of treated patients, while no grade 4–5 neurotoxicity was reported. These findings demonstrate the biological activity of bivalent CAR-T-cell therapy while also highlighting the challenges associated with treatment durability and neurological toxicity in recurrent GBM.

## 7. Conclusions

Glioblastoma (GBM) remains the most aggressive primary brain tumour, characterised by poor survival outcomes and a persistent resistance to conventional therapies. As discussed throughout this paper, its progression is driven by the dysregulation of several highly conserved signalling pathways, particularly PI3K/AKT/mTOR, MAPK/ERK, and Hippo, which collectively promote tumour growth, invasion, and therapeutic resistance. The effectiveness of current treatments is further limited by the extensive intratumoural heterogeneity and cellular plasticity that define GBM. These features enable tumour cells to adapt to therapeutic pressures through transitions between distinct transcriptional states, including the therapy-resistant mesenchymal phenotype. In addition, the blood-brain barrier (BBB) remains a major obstacle to successful treatment, restricting drug penetration and reducing the efficacy of many systemic therapies. Emerging approaches, including lipid nanoparticle (LNP)-based delivery systems, offer a promising means of enhancing drug transport across the BBB and improving therapeutic outcomes.

Evidence from clinical trials has demonstrated that targeted monotherapies, such as EGFR and mTOR inhibitors, have achieved limited success, largely because of pathway redundancy, compensatory signalling mechanisms, and the adaptive nature of GBM cells. Consequently, future therapeutic strategies are likely to depend on rational combination approaches that target multiple hallmarks of the disease simultaneously. Advances in immunotherapy have provided particular encouragement, with bivalent CAR-T-cell therapies targeting EGFRvIII and IL-13Rα2 showing evidence of meaningful tumour regression and prolonged disease stability in early studies. These developments represent an important step towards more personalised and biologically informed treatment strategies. Ultimately, meaningful improvements in patient outcomes will require an integrated approach that combines precise molecular targeting, innovative methods of overcoming the BBB, and therapies capable of reshaping the immunosuppressive tumour microenvironment. Addressing these interconnected challenges offers the greatest potential for achieving durable tumour control and improving the prognosis of patients with GBM.

## Figures and Tables

**Figure 2:**
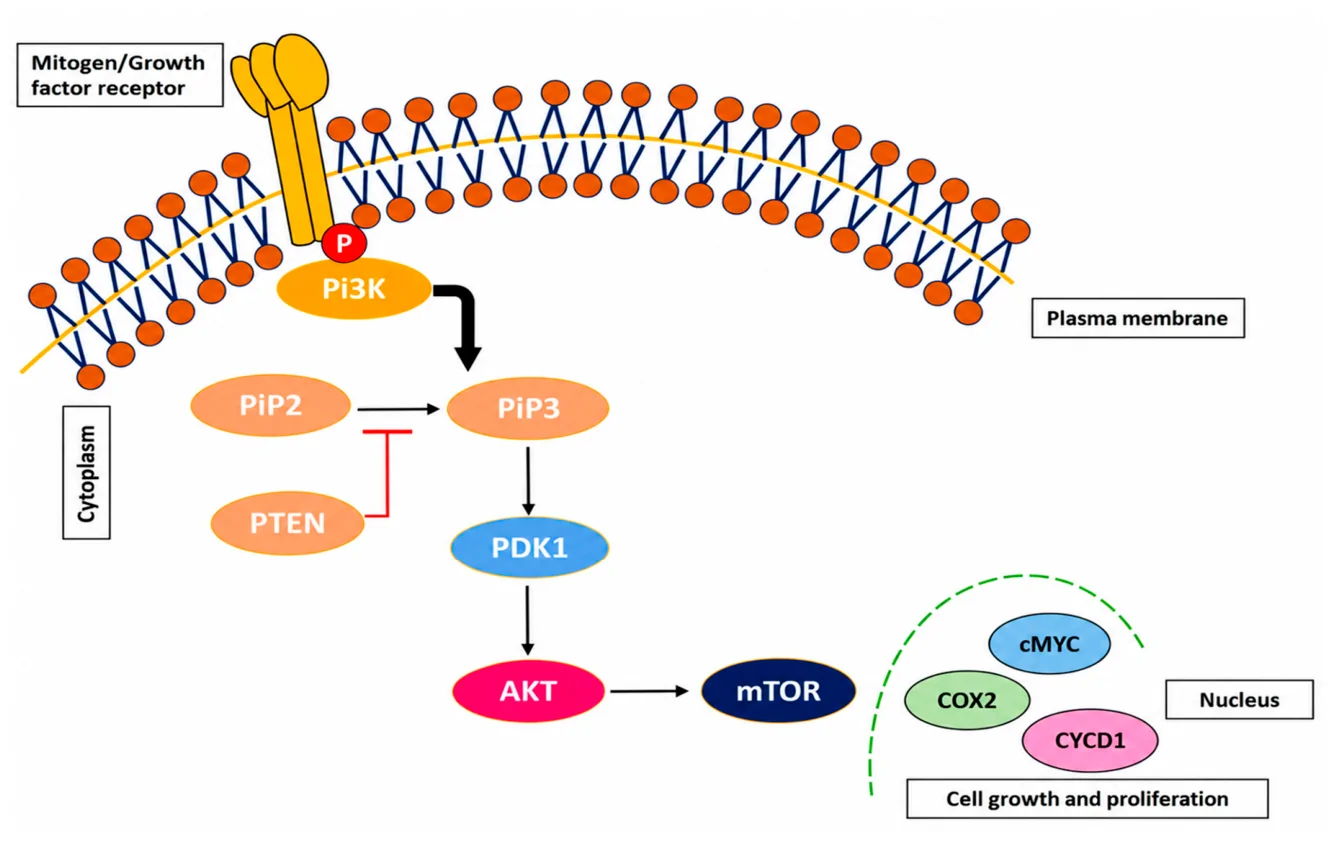
Schematic representation of the PI3K/AKT/mTOR signalling pathway. Activation of cell surface receptors, including receptor tyrosine kinases such as EGFR, stimulates Class I PI3K, comprising regulatory p85 and catalytic p110 subunits. Activated PI3K converts phosphatidylinositol-4,5-bisphosphate (PIP_2_) into phosphatidylinositol-3,4,5-trisphosphate (PIP_3_), which recruits PDK1 and AKT to the plasma membrane through their pleckstrin homology domains. Activated AKT subsequently promotes downstream signalling through mTOR and influences nuclear and cell cycle regulators, including c-MYC, CDC2 (CDK1), and Cyclin D1, thereby supporting transcriptional programmes associated with cell growth, proliferation, cell cycle progression, and survival. PTEN negatively regulates the pathway by converting PIP_3_ back to PIP_2_, thereby restricting AKT activation and downstream signalling. Taken from [26].

**Figure 3:**
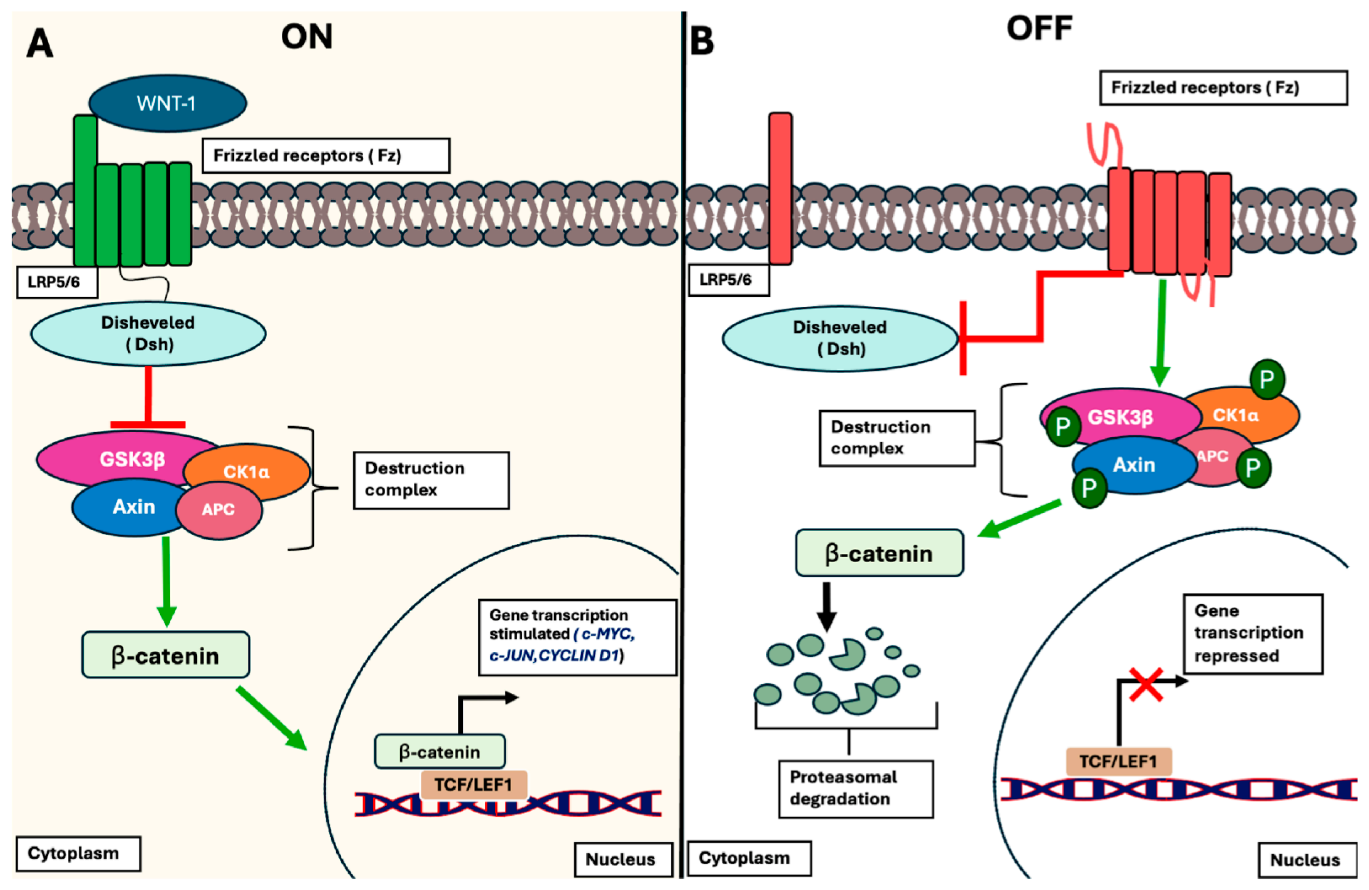
Schematic overview of the canonical Wnt/β-catenin signalling pathway. (**A**) In the absence of Wnt ligands, the pathway remains in the “OFF” state. Without activation of Frizzled (Fz) and LRP5/6 receptors, Dishevelled (Dsh) is not recruited, allowing the β-catenin destruction complex, comprising APC, Axin, PP2A, GSK3β, and CK1α, to phosphorylate β-catenin and promote its proteasomal degradation. Consequently, β-catenin-dependent TCF/LEF transcription is suppressed, indicated by the red cross. (**B**) In the “ON” state, Wnt binding to Fz and LRP5/6 promotes Dsh recruitment and inhibits destruction complex activity. β-catenin therefore accumulates in the cytoplasm, translocates to the nucleus, and associates with TCF/LEF to activate transcription of downstream targets, including C-MYC, C-JUN, and Cyclin D1, indicated by the black arrow. Taken from [26].

**Figure 4:**
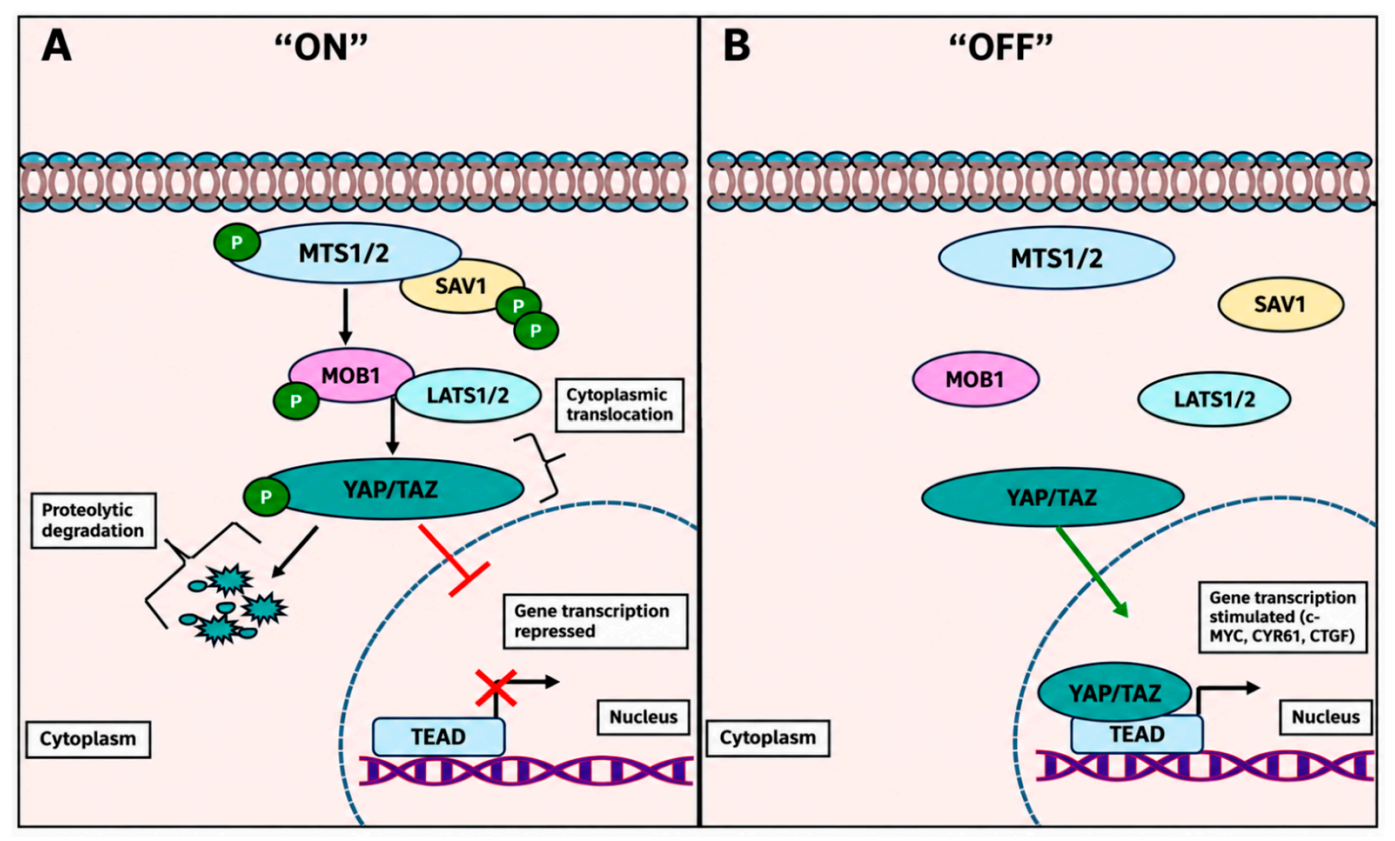
Outline of the Hippo signalling pathway. (**A**) The Hippo signalling pathway is considered in the “ON” state when MTS1/2 is phosphorylated and binds to the respective SAV1 C-domain. This allows a heterodimer to be formed, causing consecutive phosphorylation of MOB1 and LATS1/2 kinases. This results in YAP/TAZ being phosphorylated and recruiting the 14-3-3 protein, causing either cytoplasmic retention or proteolytic degradation. Thus, TEAD ultimately inhibits the transcription of target genes associated with cell proliferation, survival, and migration (perceived in red X). (**B**) The Hippo signalling pathway is in the “OFF” state when no phosphorylation of MTS1/2, SAV1-C domain, MOB1, and LATS1/2 kinases occurs. Therefore, YAP/TAZ is not phosphorylated and is localized to the nucleus, where it binds and forms a complex with TEAD to stimulate transcription of target genes such as *c-MYC*, *CYR61*, and *CTGF* (represented by a black arrow). Taken from [30].

## Data Availability

No new data were created or analyzed in this study. Data sharing is not applicable to this article.

## Abbreviations

AKT	Protein kinase B (AKT)
APC	Adenomatous polyposis coli
BBB	Blood–brain barrier
CAR-T	Chimeric antigen receptor T-cell
CD4+	Cluster of differentiation 4-positive T cell
CDC2/CDK1	Cell division cycle protein 2/Cyclin-dependent kinase 1
CK1α	Casein kinase 1 alpha
c-JUN	Jun proto-oncogene AP-1 transcription factor subunit
c-MYC	MYC proto-oncogene, basic helix-loop-helix transcription factor
CNS	Central nervous system
DNA	Deoxyribonucleic acid
Dsh	Dishevelled
EGFR	Epidermal growth factor receptor
EGFRvIII	Epidermal growth factor receptor variant III
ERK	Extracellular signal-regulated kinase
ERK1/2	Extracellular signal-regulated kinases 1 and 2
ERK/MAPK	Extracellular signal-regulated kinase/mitogen-activated protein kinase pathway
Fz	Frizzled receptor
GSK3β	Glycogen synthase kinase 3 beta
Hedgehog	Hedgehog signalling pathway
Hippo	Hippo signalling pathway
IDH	Isocitrate dehydrogenase
IL-6	Interleukin-6
IL-13Rα2	Interleukin-13 receptor subunit alpha-2
JNK	c-Jun N-terminal kinase
LATS1/2	Large tumour suppressor kinases 1 and 2
LRP5/6	Low-density lipoprotein receptor-related proteins 5 and 6
MAP	Mitogen-activated protein
MAP3K	Mitogen-activated protein kinase kinase kinase
MAP4K	Mitogen-activated protein kinase kinase kinase kinase
MAPK	Mitogen-activated protein kinase
MAPKAPK	Mitogen-activated protein kinase-activated protein kinase
MAPKK	Mitogen-activated protein kinase kinase
MEK1/2	Mitogen-activated protein kinase kinases 1 and 2
MEK/ERK	Mitogen-activated protein kinase kinase/extracellular signal-regulated kinase pathway
MOB1	MOB kinase activator 1
MST1/2	Mammalian STE20-like protein kinases 1 and 2
mTOR	Mechanistic target of rapamycin
Notch	Notch signalling pathway
p53	Tumour protein p53
PD-1	Programmed cell death protein 1
PD-L1	Programmed death-ligand 1
PDK1	3-phosphoinositide-dependent protein kinase 1
PI3K	Phosphoinositide 3-kinase
PI3K/AKT/mTOR	Phosphoinositide 3-kinase/AKT/mechanistic target of rapamycin pathway
PIP2	Phosphatidylinositol 4,5-bisphosphate
PIP3	Phosphatidylinositol 3,4,5-trisphosphate
PP2A	Protein phosphatase 2A
PTEN	Phosphatase and tensin homolog
RAS	Rat sarcoma small GTPase
RAS-RAF	RAS–RAF signalling axis
RNA	Ribonucleic acid
SAV1	Salvador family WW domain-containing protein 1
SNX7	Sorting nexin 7
TAZ/WWTR1	Transcriptional co-activator with PDZ-binding motif/WW domain-containing transcription regulator 1
TCF/LEF	T-cell factor/lymphoid enhancer-binding factor
TEAD	TEA domain transcription factor
Wnt	Wingless-related integration site signalling pathway
WNT1	Wnt family member 1
Wnt/β-catenin	Wnt/β-catenin signalling pathway
YAP	Yes-associated protein
